# Multivariate Bayesian decoding of single-trial event-related fMRI responses for memory retrieval of voluntary actions

**DOI:** 10.1371/journal.pone.0182657

**Published:** 2017-08-04

**Authors:** Dongha Lee, Sungjae Yun, Changwon Jang, Hae-Jeong Park

**Affiliations:** 1 Faculty of Psychology and Education Sciences, University of Coimbra, Coimbra, Portugal; 2 Center for Systems and Translational Brain Sciences, Institute of Human Complexity and Systems Science, Yonsei University, Seoul, Republic of Korea; 3 BK21 PLUS Project for Medical Science, Yonsei University College of Medicine, Seoul, Republic of Korea; 4 Department of Nuclear Medicine, Yonsei University College of Medicine, Seoul, Republic of Korea; 5 Department of Cognitive Science, Yonsei University, Seoul, Republic of Korea; Centre de neuroscience cognitive, FRANCE

## Abstract

This study proposes a method for classifying event-related fMRI responses in a specialized setting of many known but few unknown stimuli presented in a rapid event-related design. Compared to block design fMRI signals, classification of the response to a single or a few stimulus trial(s) is not a trivial problem due to contamination by preceding events as well as the low signal-to-noise ratio. To overcome such problems, we proposed a single trial-based classification method of rapid event-related fMRI signals utilizing sparse multivariate Bayesian decoding of spatio-temporal fMRI responses. We applied the proposed method to classification of memory retrieval processes for two different classes of episodic memories: a voluntarily conducted experience and a passive experience induced by watching a video of others’ actions. A cross-validation showed higher classification performance of the proposed method compared to that of a support vector machine or of a classifier based on the general linear model. Evaluation of classification performances for one, two, and three stimuli from the same class and a correlation analysis between classification accuracy and target stimulus positions among trials suggest that presenting two target stimuli at longer inter-stimulus intervals is optimal in the design of classification experiments to identify the target stimuli. The proposed method for decoding subject-specific memory retrieval of voluntary behavior using fMRI would be useful in forensic applications in a natural environment, where many known trials can be extracted from a simulation of everyday tasks and few target stimuli from a crime scene.

## Introduction

Decoding brain states using functional magnetic resonance imaging (fMRI) has long been applied in various research areas; for example, fMRI is used to identify explicit responses in vision [1, 2] and motor function [3] and to classify implicit brain states such as mental imagery [4], emotion [5], visual attention [6], and memory [7, 8]. Most of these studies have used block-design experiments that are more sensitive for classification than for single trial analysis. However, presentation of the same stimuli in blocks is less relevant to everyday life experiences and may lead to unwanted priming effects or intentional regulations, limiting brain decoding in certain practical environments such as forensic investigation.

For instance, let us consider a case of forensic investigation in which an investigator has photos from a crime scene. The purpose of the investigation is to identify whether the suspect voluntarily engaged in the crime or saw the scene in the picture by accident. In this case, we can design an experiment by asking participants to conduct everyday tasks (such as visiting a coffee shop and freely deciding how to act) with video recording, which will be presented during a memory retrieval task in an fMRI. These everyday tasks can then be used to localize individualized memory retrieval processes for voluntary actions versus retrieval of memory after passively seeing a video of others’ actions. In this case, we have a sufficient number of known targets, which, though not replicating the real crime scene, are similar in that they reflect actual voluntary behavior as a series of episodic memories. Here, we focus on classifying the brain responses of a single (or a few) episode(s) of target memory retrieval processes for a given cue, rather than the memory content itself, to differentiate voluntary experience from passive experience (e.g., lie detection) using fMRI. This technique operates on the assumption that voluntary action memory may be easily retrieved by spontaneous reconstruction of episodic memory as a constructive process [9, 10], while passively watching scenes may lead to incomplete recollection or memory failure [11].

Under this scenario, we propose a framework of single (or few) trial-based classification of rapid event-related fMRI signals with many known stimulus trials (acquired experimentally to localize individual brain responses to similar types of target events as described above) but few unknown target stimulus trials (e.g., real case). Many known trials are used to model brain responses and to test the unknown trials based on the model.

**Fig 1:**
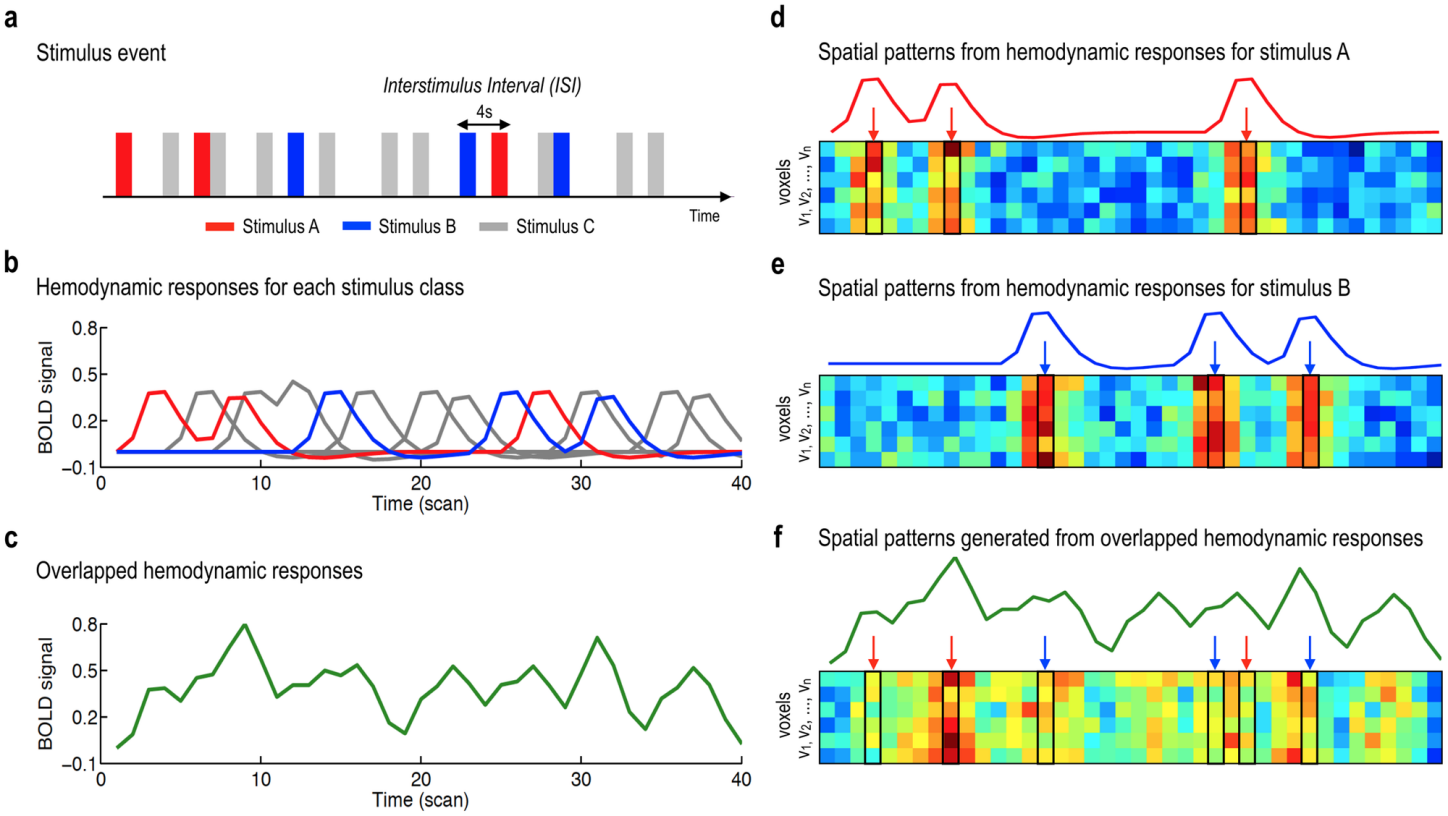
An illustration for overlapping effects of hemodynamic responses on the spatial patterns. (a) The illustration is based on the rapid event related design with three classes of events, with short intervals between events. (b) Each stimulus event elicits a class-specific hemodynamic response. (c) Due to a long hemodynamic response for a neural event, overlapped hemodynamic responses of preceding events are generally observed at each time point in the rapid event-related design. (d, e) The intrinsic neural responses can construct class-specific spatial patterns for each event, (f) whereas the overlapped responses contaminated event-specific spatial patterns.

Despite observed fMRI responses to many known stimuli, classification of fMRI responses from a single trial is not trivial, not only because of a low signal-to-noise ratio and high trial-by-trial signal variation, but also because of contamination from a previous event. Since hemodynamic responses are very slow, with a peak around 6 seconds after the neural event, the spatial pattern of blood-oxygenation-level-dependent (BOLD) signals at a time point is spoiled by the previous event (see Fig 1), which makes it difficult to classify the event. Furthermore, the low number of trials, thus the limited information available, is another bottle neck in the classification because the number of voxels used for training a classifier will highly exceed the number of scans, leading to a high dimensionality problem.

To solve those problems, the proposed method utilizes a multivariate linear model of fMRI, which models neuronally induced but hemodynamically mediated continuous “temporal” BOLD changes. This is particularly important in analyzing responses of a single stimuli with few observed data. Thus, the information from temporal dynamics should be included in the classification to compensate for the small amount of data available.

To invert the model parameters, we used a multivariate Bayesian optimization to resolve the ill-posed many-to-one mapping (many voxels to small scans or samples) [12]. The multivariate Bayesian optimization has been widely used to invert functional models involved in generating observed signals for reward-related areas [13], hippocampal subfields [14], aging differences in the formation of episodic memory [15], and motor-related areas [16] by appropriately weighting brain regions to represent target function. In this study, brain responses for a trial were modeled using multivariate Bayesian models (we denote it as MVB, hereafter), under a general linear model (GLM) of BOLD time series, and then classified via a model comparison process, i.e., selecting a better model to explain observed signals.

Effectively, this multivariate Bayesian procedure can be regarded as estimating the second order statistics of the unknown weight parameters (**β**). This can be variously interpreted in terms of covariance component estimation of the sort used in EEG source reconstruction [17,18]. Alternatively, in a machine learning setting, this can be seen as a form of Gaussian process modelling. Crucially, in both instances, sparsity is introduced into the forward or generative model through empirical spatial priors on voxel weights that ensure there are a large number of distributed responses or patterns with a very small variance and a small number with a large variance. The variance parameters correspond to the posterior distribution over voxel weights, particularly the expression of spatial patterns that encode voxel weights.

The feasibility of the proposed method in the classification of one or two target trials in the rapid event-related design was evaluated in an example of episodic memory retrieval of a voluntary experience compared to memory of a passive experience formed after seeing others’ actions. We compared the accuracy of the MVB with those of classifiers based on the GLM and support vector machine (SVM). We also evaluated the classification performance utilizing one, two, or three stimulus trials in the same class, which is feasible in a practical forensic environment. Finally, the performance dependency on target stimulus location was evaluated with respect to the inter-stimulus interval (ISI) to propose a guideline for designing target stimulus positions among known stimuli in the randomized setting for better classification in forensic application.

## Materials and methods

### Participants

The data used in the current study were part of a study presented in another paper [submitted]. In brief, 19 healthy subjects (8 males and 11 females, ages from 21 to 32 years, mean = 25.4, SD = 3.9 years) participated in the study. To evaluate all the classification methods with a same data set, we further excluded two participants who did not show increased activations in the memory retrieval of passive experience than voluntary experience, which was used as features in the generalized linear classifier. This study was approved by the Institutional Review Board of Severance Hospital, and written informed consent was obtained from all subjects.

### Experimental design

All participants visited twice with about a 10-day gap (mean 7.06 ± 3.01 days) between visits. During the first visit, participants conducted everyday tasks to create episodic memory, and during the second visit, they conducted a memory recall test in the fMRI.

#### Step 1: Formation of voluntary and passive episodic memories

On the first visit, participants chose and conducted six out of twelve everyday tasks to generate their own voluntary episodic memories such as visiting a coffee shop, ordering a drink, and asking questions to the clerk, all of which were recorded using an action video camera (GoPro HERO3 camera, https://gopro.com). After performing all field tasks, participants wrote what they did in the tasks. To create passive memories, participants also watched video clips of the other participants’ activities during their first visit.

#### Step 2: Generation of visual stimuli

To generate visual stimuli for fMRI memory retrieval task, we captured pictures from each participant’s video recording according to his/her report. The captured pictures were categorized into three different memory types: 1) 80 pictures of voluntary active experience extracted from the self-video recording, 2) 80 pictures of involuntary passive experience extracted from others’ video recordings, and 3) 80 pictures unfamiliar to the participant. To help the retrieval process, we added keywords to the pictures. For voluntary experience condition, we extracted keywords from participants' self-reports and, for passive experience condition, from the self-reports of participants chosen for others’ video recordings, and for the no experience condition, from the content materials themselves. Among different conditions, this study focused on classification of the memory retrieval process for voluntary experience and passive experience, with pictures and keywords as cues, in order to identify agency (i.e., volitional versus passive) from the target stimuli.

#### Step 3: fMRI task procedure

On the second visit, participants performed memory retrieval for a series of given cues (pictures and keywords) in the fMRI. Forty stimuli (cues) per condition were presented to participants (voluntary active experience, passive experience, and unfamiliar stimuli in 2 conditions: pictures with keywords and without keywords, total 240 stimuli) and each stimulus was presented for 2s. During fMRI scanning, participants watched and judged whether each stimulus belonged to "saw" or "didn't see" categories by pressing a button. The visual task for memory retrieval was designed using a rapid event-related design including Optseq [19] and Psychtoolbox-3 (http://psychtoolbox.org/). A cross-fixation point followed the stimulus and lasted for 1–10 s as a jitter. In order to induce memory retrieval, we were careful to ensure participants were unaware of the aim of the visual task; thus, identifying voluntariness for the stimuli involved choosing between “saw” and “didn’t see” rather than “did” and “did not do.”

### Data acquisition and image processing

Functional magnetic resonance imaging (fMRI) data were acquired axially using T2* weighted single shot echo planar imaging (EPI) sequences using a 3.0 Tesla MRI scanner (Philips Achieva; Philips Medical System, Best, The Netherlands): voxel size, 1.72×1.72×4.0 mm3; slice number, 34 (interleaved); recon matrix, 128×128; flip angle, 90°; slice thickness, 3.5 mm; slice gap, 0.5 mm; repetition time (TR), 2000 ms; echo time (TE), 30 ms; and field of view, 220 mm. To facilitate spatial normalization, a high-resolution structural data set was obtained from each subject with a SENSE head coil using a 3D T1-TFE sequence configured with the following acquisition parameters: axial acquisition with a 224 × 224 matrix, 220 mm field of view, 0.98 × 0.98 × 1.2 mm voxel unit, 4.6 ms TE, 9.6 ms TR, 8° flip angle, and 0 mm slice gap.

FMRI data preprocessing was conducted using statistical parametric mapping (SPM12, http://www.fil.ion.ucl.ac.uk/spm/, Wellcome Trust Centre for Neuroimaging, London, UK) [20]. After discarding the first 5 scans to address the stability issues, all EPI data underwent preprocessing steps including correction of the acquisition time delay between different slices, and correction for head motion by realigning all consecutive volumes to the first image of the session. The realigned images were co-registered to T1-weighted images, which were used to spatially normalize functional data into a template space using nonlinear transformation. The normalized data were spatially smoothed with a 6-mm full-width-at-half-maximum (FWHM) isotropic Gaussian kernel.

Considering forensic application, we mainly focused on classifying BOLD responses to stimuli (pictures with keywords) from voluntary active experiences and involuntary passive experiences. In this respect, we designed contrasts between active and passive experiences to analyze the fMRI data. To extract task-elicited activation regions as initial feature masks for each individual, we conducted statistical parametric mapping of BOLD signals in the rapid event-related design using SPM12. Voxel clusters comprising a minimum of 35 consecutive voxels with statistical value over a threshold of p < 0.001 (uncorrected) were chosen for each condition (voluntary and passive experience) in the individual activation map. We combined activation maps of both voluntary and passive experience (VE and PE) to create a feature mask for the subsequent analysis. To evaluate a better feature mask in the classification, we also generated a feature mask composed of voxels showing the statistical difference between voluntary and passive experiences (VE—PE). In this case, we initially applied a statistical threshold of p < 0.001 (uncorrected) with 35 consecutive voxels but adjusted the threshold level to include voxels related to voluntary, passive experiences, or both.

### Multivariate Bayesian parameter estimation and basic concept for classification

To infer brain states from fMRI signals, we proposed two MVB models explained in the following section, utilizing the multivariate Bayesian model parameter estimation (MVBPE) in the SPM toolbox. Although the details of the multivariate Bayesian model inversion method can be found in Friston, Chu (12), the method and procedures are briefly described below.

A decoding model is based on the reversal of the conventional GLM, where observed signals are represented by a design matrix **X** and its weight vector **β**. In the MVB, a scalar target variable *X* ∈ *R* represents a scan-specific measure of behavioral state and corresponds to a linear mapping of a voxel intensity vector (number of voxels N), $A=\left\{A_{1}^{t},\;A_{2}^{t},\;\ldots ,\;A_{N}^{t}\right\}$ at scan time *t* = 1, …, *M* and voxel weights **β** = (*β*_1_, …, *β*_*N*_), defined below:

X=Aβ (1)

To include hemodynamic response effects, a temporal convolutional matrix *T* with an embedded hemodynamic response function was multiplied to Eq (1).

TX=TAβ (2)

Observed fMRI data {**Y**_*t*_}_*t* = 1, …, *M*_ correspond to intensities **Y**_*t*_(**v**) at scan time *t* at voxels **v** = {*v*_*i* = 1, …, *N*_|*v*_*i*_ ∈ *ROI* within a localized brain region (ROI) in the initial feature mask. fMRI responses **Y** can then be represented by the sum of *T***A**, confounds *G* scaled by unknown parameters *γ*, and noise *ε*.

Y=TA+Gγ+ε (3)

By combining Eqs (2) and (3), the following can be derived:

TX=TAβ=Yβ−Gγβ−εβ (4)

where *TX* can be written by a combination of regressors for events of multiple classes and a contrast weight vector *c*, *TX =* **X***c*, which corresponds to the subspace of the design matrix

$$X=\left[\begin{array}{cccc} X_{11} & X_{12} & \cdots & X_{1J}\\ X_{21} & X_{22} & \cdots & X_{2J}\\ \vdots & \vdots & & \vdots \\ X_{M1} & X_{M2} & \cdots & X_{MJ} \end{array}\right]$$

for M scans and J regressors.

By applying a residual forming matrix, *R* = *orth*(*I* − *GG*^−^)^*t*^, to remove the confounds from the model, we can have a simplified model for the scalar target variable *X* weighted by a weighting matrix *W*.

WX=RTX=RYβ+ς,  ς=−Rεβ (5)

where *ς* are a vector of unknown random effects, following multivariate Gaussian distribution with covariance, Σ^*ς*^ = exp(*λ*^*ς*^)*RVR*^*T*^. Here, *λ*^*ς*^ is a hyperparmeter and *V* represents serial correlations.

To make the ill-posed regression problem tractable, empirical priors on voxel weights **β** can be embedded by introducing a second level in the model.

β=Uη (6)

where *U* contains spatial patterns to impose constraints and *η* are unknown pattern weights as second-level random effects with covariance, Σ^*η*^. We can model the covariance matrix Σ^*η*^ as a linear combination of *m* covariance components (or leading diagonal matrices, *I*^(1)^, …, *I*^(m)^: $\Sigma^{\eta }=\text{exp}\left(\lambda_{1}^{\eta }\right)I^{\left(1\right)}+\text{ exp}\left(\lambda_{2}^{\eta }\right)I^{\left(2\right)}+\cdots +\text{exp}\left(\lambda_{m}^{\eta }\right)I^{\left(m\right)}$. This exerts empirical priors on the voxel weights **β**, *p*(**β**) = *N*(0, *U*Σ^*η*^*U*^*T*^). In the current study, we used spatial patterns, *U* = *I* to impose a sparse constraint.

The two-level model represented in Eqs (5) and (6) can be combined into a single question:

v=WX=RYUη+ς=Lη+ς,    L=RYU (7)

The covariance of *v* can be written:

$$\Sigma^{v}=\Sigma \left(\lambda \right)=L\Sigma^{\eta }L^{T}+\Sigma^{\varsigma }=\text{exp}\left(\lambda_{1}\right)Q_{1}+\text{ exp}\left(\lambda_{2}\right)Q_{2}+\cdots +\text{exp}\left(\lambda_{m+1}\right)Q_{m+1}$$ (8)

$$\lambda =\left\{\lambda^{\varsigma },\lambda_{1}^{\eta },\ldots ,\lambda_{m}^{\eta }\right\}$$

$$Q=\left\{RVR^{T},LI^{\left(1\right)}L^{T},\ldots ,LI^{\left(m\right)}L^{T}\right\}$$

Since only hyperparameters *λ* are unknown in this equation, the decomposition of covariance significantly reduces the number of parameters to estimate.

To find a posterior distribution over voxel weights **β**, a Bayesian model inversion with expectation maximization optimization algorithm (EM) was used to fit the model by maximizing free-energy, F, defined under Laplace approximation as below [12].

$$\text{ln}p\left(\text{X}|\text{Y}\right)\geq \text{F}=-\frac{1}{2}\left(X^{t}W^{t}\Sigma {\left(\mu^{\lambda }\right)}^{-1}WX-\text{ln}\left|\Sigma \left(\mu^{\lambda }\right)\right|-w\text{ln}2\pi +\text{ln}\left|\Pi \Sigma \lambda \right|-{\left(\mu^{\lambda }-\pi \right)}^{t}\Pi \left(\mu^{\lambda }-\pi \right)\right),$$ (9)

where *X* comprises the target variables and *W* is the (known) weighting matrix. Gaussian prior leads to a Gaussian likelihood (*p*(*X*|*Y*, *λ*) = *N*(0, Σ(Y, *λ*)) and is specified by a Gaussian covariance Σ(Y, *λ*) with given fMRI data Y and covariance hyperparameter *λ*. For approximation, the prior, *p*(*λ*) = *N*(*π*, Π^−1^) with the prior expectation π, and covariance Π, and posterior *q*(*λ*) = *N*(*μ*^*λ*^, Σ^*λ*^) with the conditional mean μ and covariance Σ of λ are used.

**Fig 2:**
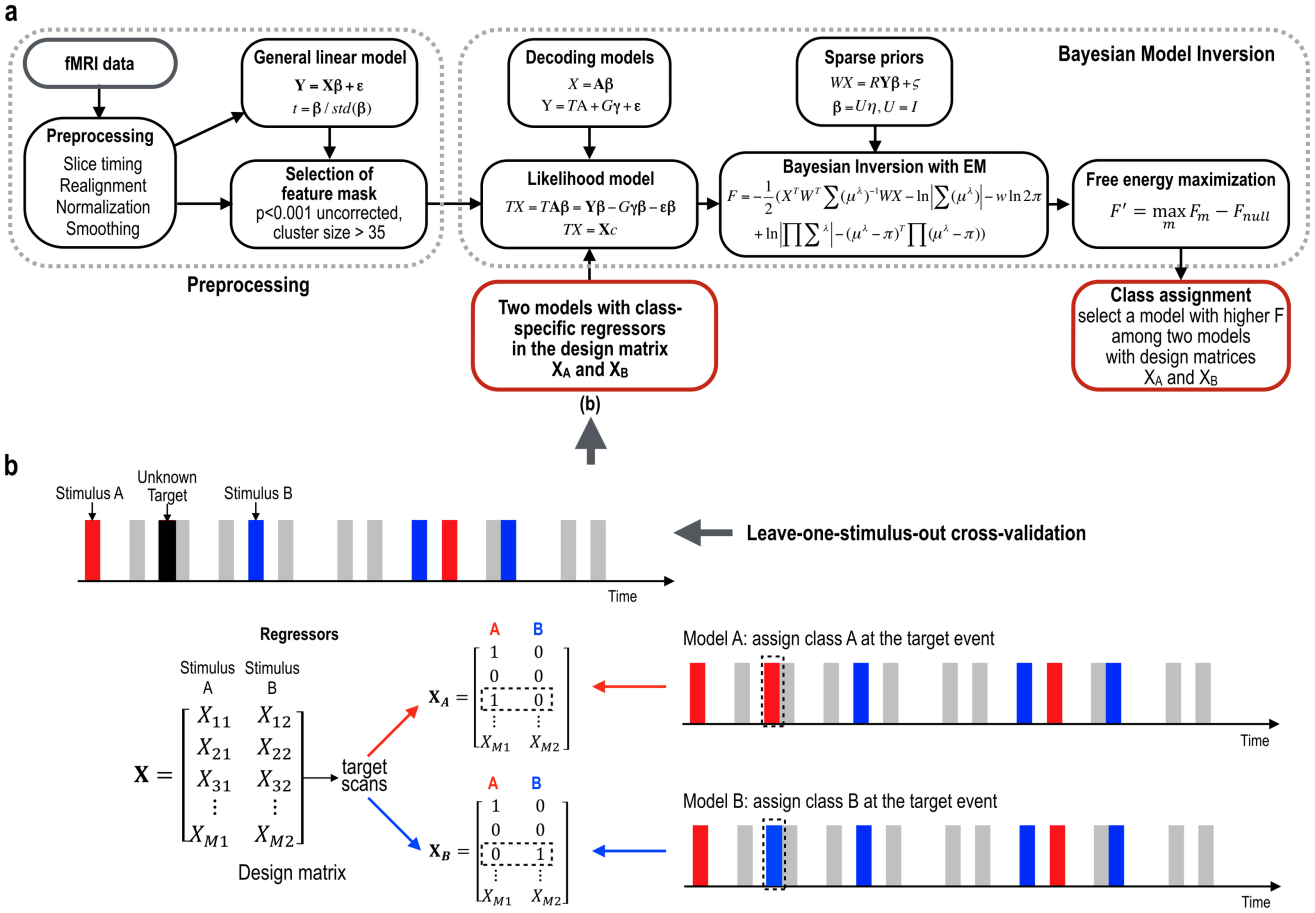
Detecting brain states using multivariate Bayesian inversion scheme. (a) A general overview for decoding. (b) An example of application in a rapid event related design to models with different design matrices X_A_ (assigning 1 for the regressor A and 0 for the regressor B at the target stimulus) and X_B_ (assigning 0 for the regressor A and 1 for the regressor B at the target stimulus) assuming the unknown target class as class A and class B, respectively. The class for the unknown stimulus was chosen by selecting the model with higher free-energy F among models with different design matrices X_A_ and X_B_.

The class for the unknown target trial with different design matrices is determined by finding a class label with the maximal Free energy approximation value among models with different design matrices. This process is summarized in Fig 2a. Fig 2b illustrates regressors with different assumptions of the class label for the unknown target in the design matrix X, which is used to determine the class for the target stimulus.

This is an efficient Bayesian model comparison procedure that compares the evidence for hypothetical target variables (e.g., voluntary or passive experience). The use of Bayesian model comparison is the innovation introduced by this paper. Originally, MVB was introduced to enable Bayesian model comparison of different spatial patterns in terms of empirical spatial priors on voxel weights (i.e., spatial coding hypothesis, *U*). However, in our application, we compare models in terms of different design matrices or target variables (i.e., *X*). This enables us to convert a classification problem into an evidence-based model comparison problem.

### MVB models for classification

**Fig 3:**
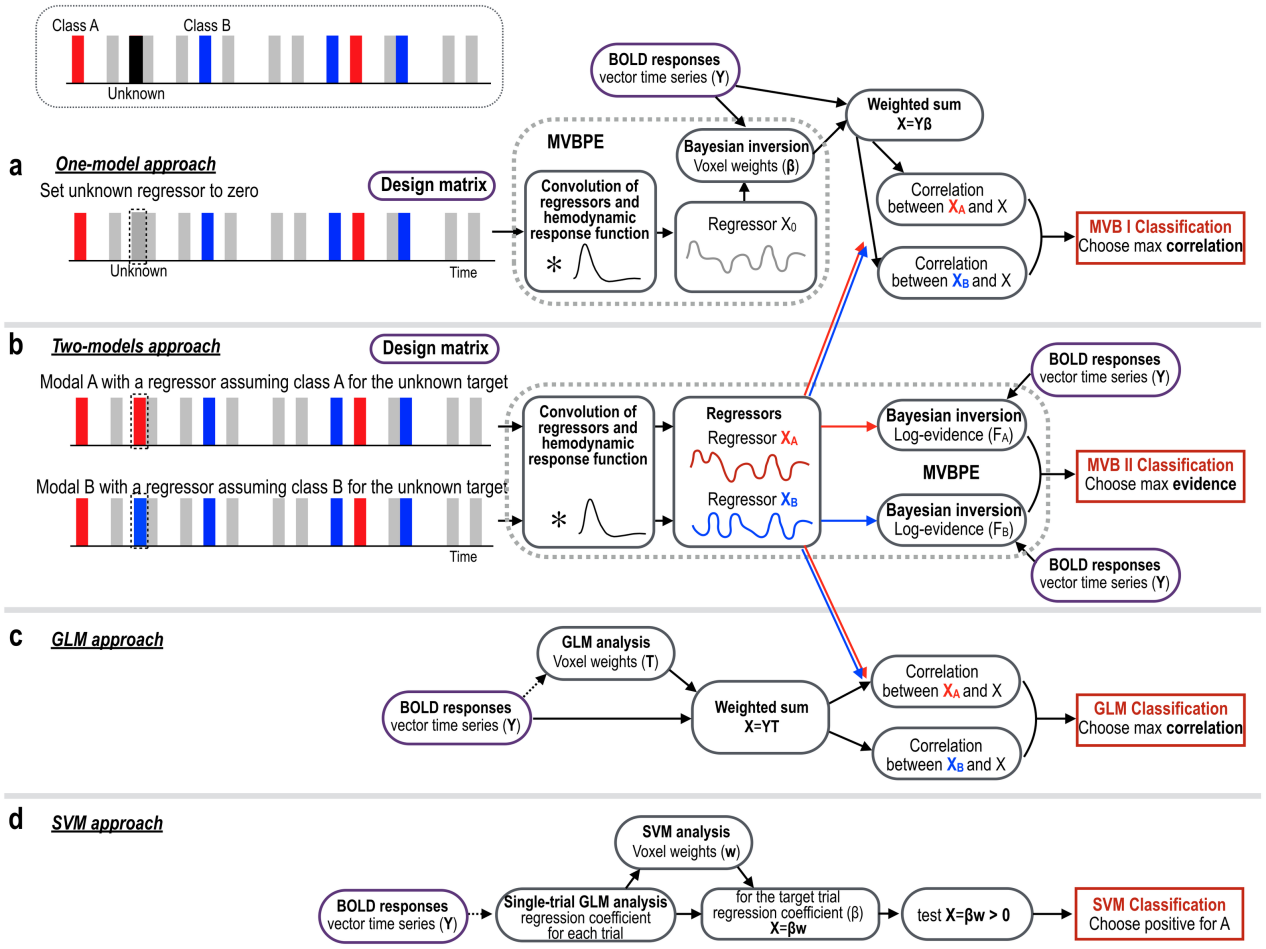
Illustration of the one-model and two-models MVB approaches. (a) To classify an unknown stimulus, the one-model method builds a MVB model with a regressor of known stimuli by ignoring the unknown targets. In this model, multivariate Bayesian parameter estimation (MVBPE) estimates model parameters, β. The weighted sum of fMRI voxel time series (weighted by the model parameters, β) was correlated with a regressor of A class (X_A_) and a regressor of B class (X_B_), then the regressor with higher correlation was chosen for the target class label. (b) Two-models approach builds two types of MVB models with two regressors with A and B classes for the target stimulus. The model with higher free-energy (estimate of log-evidence) was chosen for the target class label. This model utilizes the free-energy estimated by MVBPE instead of model parameters, β. (c) The T-value weighted sum of fMRI time series (GLM analysis) was correlated with two regressors (X_A_, X_B_). A class with higher correlation coefficient was chosen as the class for the target. (d) The class was assigned to class A when the dot-product of weights of SVM classifier (trained with single trial regression coefficients in the GLM analysis) and the regression coefficients (β) for each single stimulus was higher than zero, and otherwise class B.

In this study, we proposed a MVB-based classification method to classify single (or few)-trial(s) fMRI responses from two memory retrieval processes, voluntary and passive experiences, by utilizing the multivariate Bayesian model inversion of fMRI voxel activities in the feature mask. Note that the multivariate Bayesian model inversion method in Friston et al. [12] itself is not a classifier, rather we utilized its model comparison scheme for the classification. Based on the assumption that retrieval processes for voluntary and passive experiences are differentially manifested in neural responses, we conducted and compared two Bayesian approaches in classifying each type of memory retrieval. The first approach employed known trials to build a model and to apply it to test data sets. The second approach, a model comparison method, constructed two models after assigning a class to the target in the design matrices. The two types of approaches are explained in Fig 3.

1. **One-model approach.** We built a single model only with known stimuli-responses and inverted the model parameters β (i.e., voxel weights) using MVBPE described in the above section. More specifically, we generated a design matrix (a regressor) by assigning the target stimulus as zero as if no event occurred at the time point (Fig 3a). With this design matrix, we estimated optimal model parameters β (voxel weights) using the MVBPE with a sparsity prior, which were multiplied by the observed fMRI time series at corresponding voxels to generate a signal at the target stimulus. The correlations between the target signal and two model regressors (one assuming the missing target as voluntary experience and the other as passive experience) after hemodynamic convolution were compared, and the model with a higher correlation was assigned as the class for the unknown stimulus.
2. **Two-model approach.** In this approach, we built two MVB models: 1) a model with an assumption of voluntary experience class and 2) another model with an assumption of passive experience class for the unknown event. More specifically, we built MVB models with two different regressors for the stimuli sequence, assigning each to the voluntary experience and the passive experience for the target event (Fig 3b). We estimated optimal model parameters β and the free-energy (estimated log evidence) using MVBPE with a sparsity prior. By comparing the maximized free energy (approximated log evidence) of both models, we classified an event as the higher free energy class. In this approach, we did not use model parameters β in the classification but used free-energy associated with optimal β in the model comparison.

### GLM-based classification

The simplest way to identify missing label is to use a GLM (Y = Xβ+ε). Since the estimate of coefficient (β) reflects voxel weights for brain activity for a given class, we can interpolate the brain response corresponding to the target event by multiplying these weights with the fMRI time series.

In the GLM-based classification, the time series of voxels in the voluntary and passive contrast maps were T-value weighted and summed to generate the target variable. This target variable was correlated with two regressors (one for assuming voluntary experience and the other for assuming passive memory) convolved with hemodynamic response function. The class with the higher correlation coefficient was chosen as the class for the target (Fig 3c).

### SVM classification

We conducted SVM classification of a single trial using the Spider for MATLAB (http://people.kyb.tuebingen.mpg.de/spider/). We first estimated trial-wise beta estimates using GLM, following Mumford et al.[21]. The regression coefficient, β, of each trial was estimated by applying GLM with a regressor only for that trial (i.e., 1 for the trial and 0 for the other trials) convoluted by a hemodynamic response. Total 80 β-values (VE: 40 trials, PE: 40 trials) were used as samples in the binary SVM classification. The data were divided into a training data set (79 β-samples) and a testing data set (one β-sample) similar to leave-one-out approach. We trained a linear SVM classifier with a regulation parameter C = 1 [5, 6]. In order to reduce the feature dimension, we conducted Recursive Feature Elimination (RFE), which recursively excluded weakly informative voxels reflected in the smallest absolute weight value [22, 23]. In the initial feature mask, RFE selects features with greedy backward selection from all features using the training data by recursively removing half of weakly informative (small absolute weights) features (voxels). The process was iterated until the number of features reached about 2,000, the level associated with an average feature number in the MVB optimization (average 1,940 sparse features) (Table 1).

**Table 1:** Summary of individual features and accuracies of MVB optimization according to the number of trials.

Subjects	VE & PE				
	#initial voxels	#final features	MVB accuracy (single)	MVB accuracy (two)	MVB accuracy (three)
S1	30359	2048	80.00	70.00	85.00
S2	8284	2048	65.00	85.00	77.50
S3	27747	2048	83.75	97.50	100.00
S4	43577	1362	72.50	87.50	100.00
S5	42535	2048	83.75	82.50	86.25
S6	15754	2048	78.75	100.00	100.00
S7	75809	2048	78.75	100.00	100.00
S8	30913	1933	76.25	93.75	87.50
S9	15216	2048	61.25	75.00	67.50
S10	77760	2048	50.00	78.75	75.00
S11	78551	2048	71.25	98.75	98.75
S12	54022	2048	75.00	95.00	93.75
S13	44291	1385	72.50	87.50	83.75
S14	16225	2048	78.75	81.25	76.25
S15	8612	2048	90.00	93.75	98.75
S16	47536	2048	56.25	54.38	52.50
S17	53584	1675	53.75	52.50	51.25
Mean	39457	1940	74.49	87.57	88.31

### Comparison of classification performance

**Fig 4:**
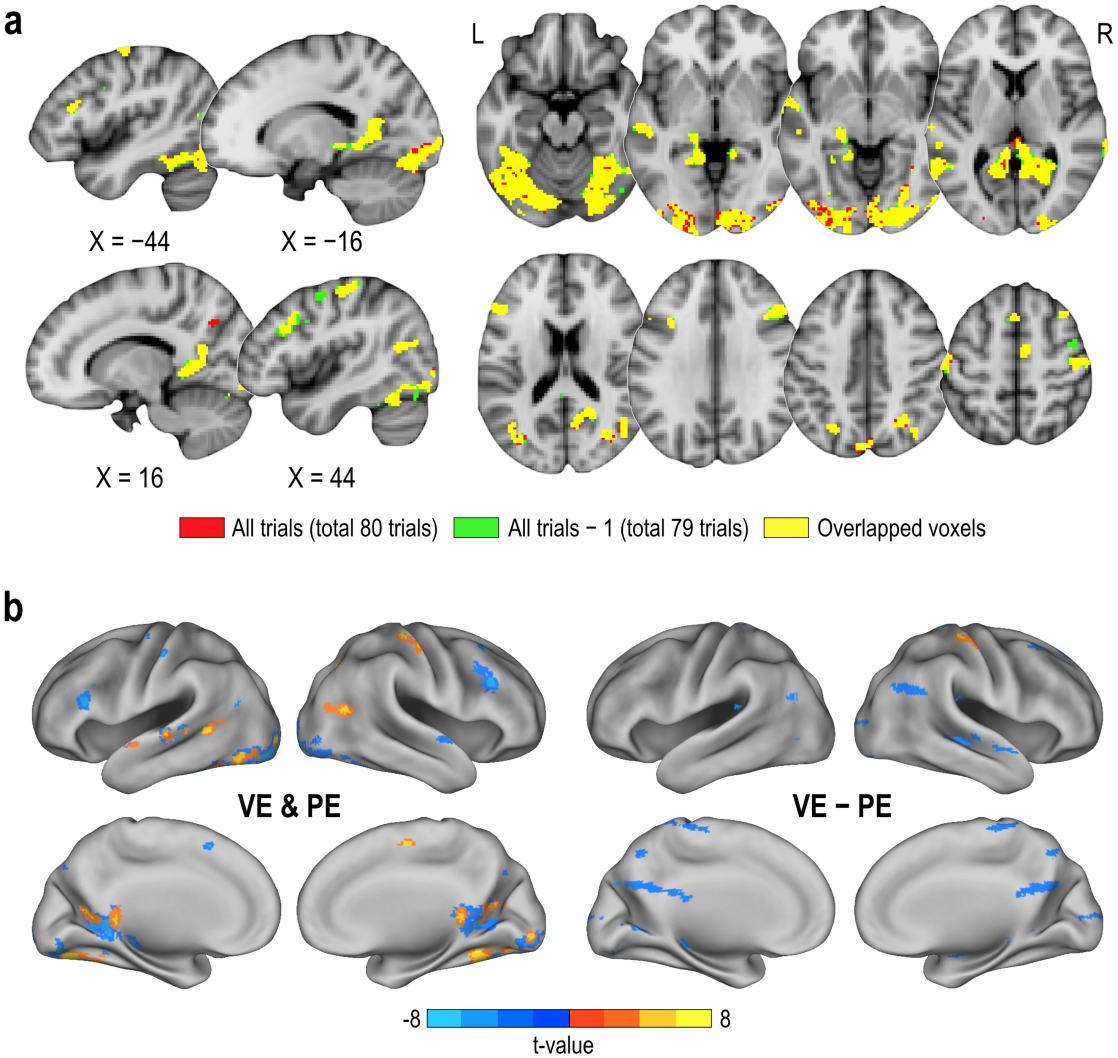
Comparison of initial feature masks for decoding. (a) The initial feature masks from statistical parametric maps generated from all trials (total 80 trials, colored in red and yellow) and all trials except for a test trial (total 79 trials, colored in green and yellow) of a participant were compared. The initial feature masks show 95% overlaps (in yellow color). (b) The initial feature masks derived from the statistical parametric maps of union and difference between voluntary experience and passive experience (VE & PE and VE − PE) in an individual participant are displayed. Red-yellow color indicates increased activation in memory retrieval of a voluntary experience than in a passive experience, while reverse for blue colors.

All processes for classification (i.e., training and testing) using the GLM, SVM, and MVB model-based classification were conducted for each individual. In theory, when evaluating the classification performance, the training data and testing data should be independent not only in model estimation but also in feature selection. In the current study, we had two levels of feature selection process; 1) initial feature mask from GLM analysis (thresholded with a supra-threshold) and 2) features after feature selection or optimization in MVB (with a sparsity prior) or SVM (using RFE). Although we used training and testing data independently for the second step, we decided to minimize the huge computational demands by choosing a feature mask of the entire time series (including testing data as well) in the evaluation of each single trial classification in this special application. This is because we assumed that statistical parametric maps generated from any combination of 79 (or 78) trials (exclusion of any unknown single (or two) test trial(s) out of 80 whole trials for VE and PE) would not be highly different from that of the whole trials (80 trials). To evaluate this assumption, we compared the feature masks of VE and PE obtained from all trials (total 80 trials) and from all trials except one condition scan (total 79 trials) of a participant using the overlap rate, i.e., the ratio of the number of intersecting voxels to that of total voxels in percentile. We found the two feature masks were highly consistent (95% overlaps) (Fig 4a).

To evaluate the performance among classification methods, we used a leave-one-out cross-validation instead of k-folds cross-validation. This is because the purpose of our current method is to classify a single target trial (two or three trials) using the other trials for model optimization, and also because BOLD time series are consecutive, which cannot be easily separated. Among 80 trials (40 for voluntary experience and 40 for passive experience), we chose a trial as an unknown target and classified this trial using the other 79 trials. This process was repeatedly done for each single trial.

We first compared the two types of sparse MVB approaches: 1) one-model and 2) two-model approaches. Of the two MVB approaches, the one showing better performance was compared with GLM-based and SVM classifications. We compared classification methods using paired t-tests of classification accuracy across all subjects.

### Performance evaluation for multiple trials

We also conducted classification for two and three trials with the same class as unknown targets using the GLM classifier, SVM, and sparse MVB. Instead of using two or three consecutive trials, we randomized the target trials to ensure that the participants did not pay special attention to the target events, which may reflect an attempt to hide their memories in practical application.

We first chose a reference trial of a condition (e.g., voluntary experience) that had the longest ISI. We then chose a trial out of the remaining 39 trials of the same class (voluntary experience) and conducted MVB optimization of the trial as described above. We repeated this process for all 39 trials and calculated the mean classification accuracy.

This was repeated for the passive experience condition. For three known stimuli, we first chose a reference trial and found the second and the third trials that had the next longest ISIs. Evaluation of classification performance of multiple trials was conducted in the same way as the two-trial classification.

### Performance evaluation for stimulus location

The performance dependency on target stimulus location with respect to pre, post and total ISI was evaluated by analyzing the correlation coefficient between the target stimulus and its free-energy. Free-energy for each trial is an approximate of log model evidence, indicating the model goodness of fit for the observed BOLD signals. In this study, classification of each trial is based on model comparison using free-energies of two different models with different design matrices. We conducted correlation analysis between free-energy differences and duration between the onset time of the preceding stimulus and that of the current stimulus (PRE-ISI), the duration between the onset time of the current stimulus and that of the following stimulus (POST-ISI), and the duration between the preceding and following stimulus onset times (TOTAL-ISI).

## Results

Fig 1 shows an example of a rapid event-related design that exhibits event-overlapping effects on both the temporal and spatial patterns of hemodynamic responses, which poses challenges in classification by using spatial patterns at a single time point (Fig 1f).

For the evaluation of the classification of each individual, we used two different types of feature masks from the individual statistical parametric maps: 1) a map showing memory retrieval of either voluntary experiences or passive experiences, and 2) a map showing statistical differences between memory retrieval of voluntary versus passive experiences (Fig 4b).

**Fig 5:**
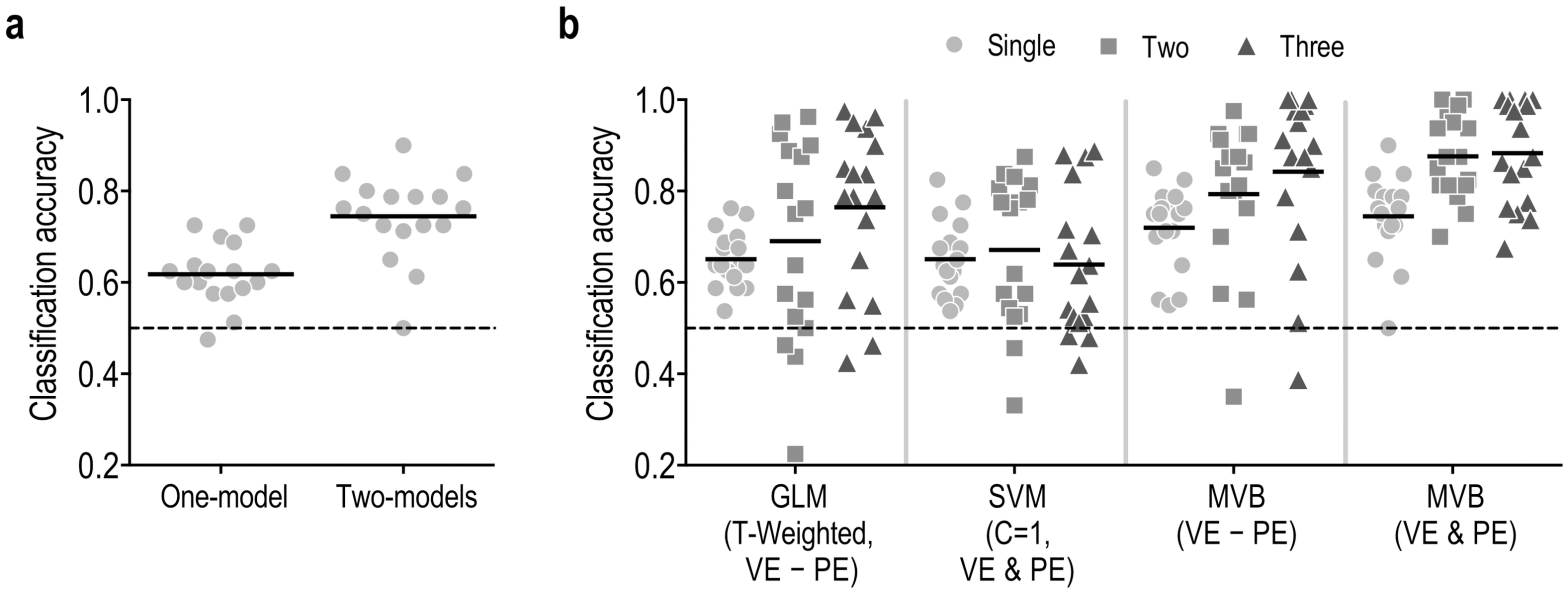
Classification accuracy of the sparse MVB compared to other classification methods. (a) In the MVB model-based classification for single trial, the two-models approach showed significantly higher accuracy than the single-model approach (p < 0.001). (b) Comparison results of MVB model-based classification performance compared to GLM and SVM for single, two and three trials are displayed. The classification accuracy of all the methods are statistically higher than the chance level of 0.5 after one sample t-tests (p < 0.05). The proposed MVB (VE & PE) method with a feature mask containing both voluntary experience (VE) and passive experience (PE) showed greater classification accuracy for single and multiple trials than the classification method based on GLM (T-weighted), SVM (the parameter C = 1 over the feature mask VE & PE), and MVB (VE—PE, over the feature mask in the contrast between voluntary experience versus passive experience).

In the GLM-based classification, classification of features in the VE—PE contrast map (voxels responding more to retrieval of voluntary memory compared to passive memory) showed significantly higher performance than those in the VE & PE contrast map (voxels responding to retrieval of either voluntary or passive experience) (one trials: VE−PE = 0.65 ± 0.06 (t_(16)_ = 10.11, p < 0.001), VE & PE = 0.51 ± 0.03, (t_(16)_ = 1.83, p = 0.0864), paired t-test: p = 4.3e-08; two trials: VE−PE = 0.69 ± 0.22 (t_(16)_ = 3.60, p = 0.0024), VE & PE = 0.51 ± 0.21 (t_(16)_ = 0.27, p = 0.7910), p = 0.0446; three trials: VE−PE = 0.76 ± 0.18 (t_(16)_ = 6.22, p < 0.001), VE & PE = 0.52 ± 0.19 (t_(16)_ = 0.47, p = 0.6449), p = 0.0036). Therefore, we used the GLM-based classification with VE-PE in the comparison, shown in Fig 5b and Table 2.

**Table 2:** MVB model-based classification performance compared to GLM and SVM for single and multiple trials. * indicates that the MVB method using VE and PE (VE & PE) contrast map has significantly higher accuracy compared to the other methods at each trial (*p<0.05, **p<0.01, ***p<0.001). Mean ± Standard deviation. Accuracy in %. † indicates a tendency of difference between MVB (VE-PE) and MVB (VE & PE) (p = 0.06).

Number of trials	GLM (T-weighted, VE–PE)	SVM (C = 1, VE & PE)	MVB (VE–PE)	MVB (VE & PE)
One	**65.1 ± 6.2	**65.1 ± 8.3	72.0 ± 9.1	74.5 ± 9.3
Two	**69.0 ± 21.8	***67.1 ± 16.1	79.3 ± 16.4^†^	87.6 ± 9.2^†^
Three	*76.5 ± 17.6	***63.9 ± 15.5	84.3 ± 18.3	88.3 ± 11.2

Fig 5a shows classification results for single trial classification using one- and two-model MVB approaches. The two-model approach showed a significantly higher accuracy compared to that of the one-model approach for single trial analysis (one-model = 0.62 ± 0.07, two-model = 0.75 ± 0.09, p = 2.2e-05; Fig 5a). We therefore used the two-model approach as a default MVB method in the evaluation hereafter.

Fig 5b and Table 2 shows the classification performance of the proposed MVB method compared to those of the GLM and SVM methods for one, two, and three trials. MVB with voxels responding to retrieval of either voluntary or passive experience (VE & PE) showed the highest accuracy, in relation to the GLM and SVM procedures. In the MVB with voxels responding to either voluntary or passive memory retrieval, two- and three-stimuli trials increased classification accuracy to a significantly higher degree than the single trial (repeated ANOVA measures, p = 4.2e-07; one = 0.74 ±0.09, two = 0.88 ± 0.09, three = 0.88 ± 0.11; two > one, p = 9.0e-05; three > one, p = 1.7e-05). However, there was no significant difference in performance between two and three trials (p = 0.6983). Two trials are optimal in terms of balance between accuracy and number of trials.

**Fig 6:**
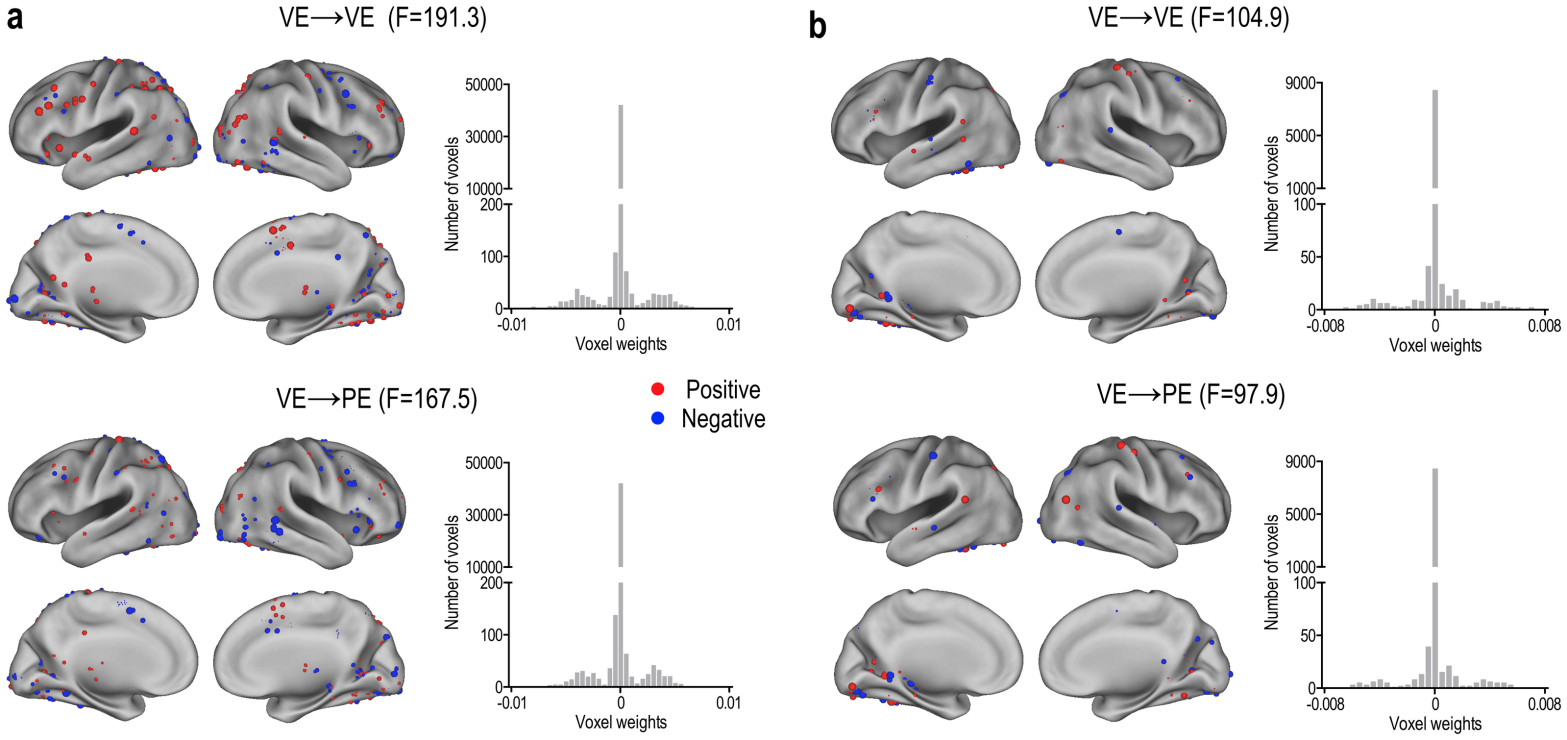
Exemplary display of distributed sparse feature maps used to decode voluntary and passive visual stimuli in two participants. The feature maps in (a) and (b) were generated based on the voxel weights from two MVB models. In these examples, model parameters (voxel weights) estimated by assuming the unknown target stimuli as either voluntary experience class (VE→VE) or passive experience class (VE→PE) are displayed, with red colors for positive weights and blue colors for negative weights. The size of spheres indicates the strength of weights. The histograms of the voxel weights (features) show small non-zero values showing sparsity.

Fig 6 displays examples of the voxel weights in two subjects with high classification accuracy for a single trial. The voxel weights for discrimination of voluntary and passive memory stimuli were distributed sparsely and differentially according to subjects (Fig 6a and 6b). Fig 6c and 6d show histograms of voxel weights in these subjects. The histogram showed a small proportion of non-zero voxel weights (sparsity) among the feature mask in the MVB.

**Fig 7:**
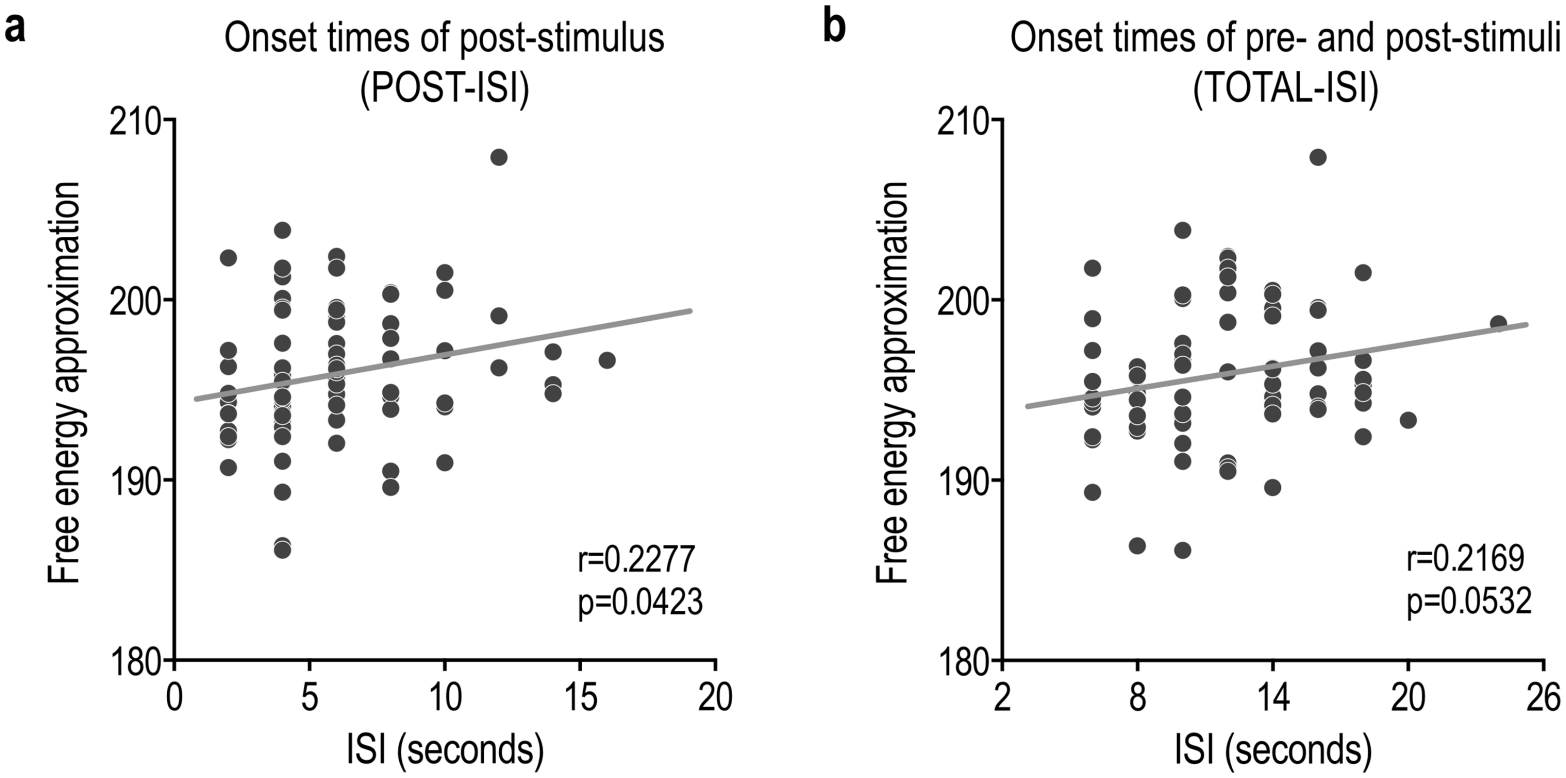
Effects of interstimulus interval on the free energy approximation. (a) The Free energy approximation had a significant positive correlation with interstimulus intervals between onset times of present and next stimulus (POST-ISI) (r = 0.2277, p = 0.0423). (b) There was a tendency toward positive correlation between the Free energy approximation and the total interstimulus interval (TOTAL-ISI) (r = 0.2169, p = 0.0532).

We evaluated the relationship between the model goodness of fit measured using free energy approximation and the position of the target stimulus compared to other stimuli with respect to ISI. Fig 7 shows the effects of ISI on free energy approximation. The free energy approximation had a significant positive correlation with the POST-ISI (r = 0.23, p = 0.0423; Fig 7a). The correlation between the free energy approximation and the TOTAL-ISI showed a tendency toward positive correlation (r = 0.22, p = 0.053; Fig 7b).

## Discussion

This study proposes a framework for trial-by-trial classification in a specialized setting with many known and few unknown stimuli in a single subject. The framework is based on a multivariate Bayesian model comparison to classify noisy hemodynamic responses of the unknown target stimuli presented in a rapid event-related design for each individual.

In contrast to many block-designed brain decoding studies using fMRI [2–5, 7, 24–28], very few studies have conducted classification of events using rapid event-related design [21, 29, 30]. Although the spatial pattern (or voxelwise activity pattern) in the block design may have higher a signal-to-noise ratio than that in event-related design, leading to a better classification performance, the block design is not as relevant in natural settings and practical applications, such as forensics. In these scenarios, an event-related design is a better choice than block design, and in particular, a rapid event-related design is more advantageous than a slow event-related design in simulating everyday life task [31].

In terms of event classification, however, a BOLD response to a stimulus in the rapid event-related design is weaker than in the block design. Furthermore, since the rapid event-related design does not have sufficient time to recover from a reaction to a stimulus, the BOLD signals at the time of a stimulus consist of overlapping responses to preceding stimuli, which makes it difficult to classify a single trial without considering temporal contexts. Therefore, to better classify hemodynamic responses for a single stimulus and to minimize the overlapping problem due to consecutive events, gaining more information by utilizing spatio-temporal data is necessary.

Previous fMRI classification studies with rapid event-related design experiments have tried diverse methods to utilize temporal information such as averaged activities of events using temporal compression [30] or beta estimates (regression coefficients) using GLM [21] for classification. In Connolly et al. [30], features were driven by the average responses for a class of stimuli. This method did not take into account the impact of previous event in the temporal model. Mumford and colleagues [21, 32] have used GLM regression coefficients in the classification with two regressors; one trial as a regressor and all the other trials as a second regressor. Janoos et al. [29] proposed a fully spatio-temporal multivariate analysis method using a state-space model and a hemodynamic response model.

In this study, two spatio-temporal multivariate Bayesian models (MVBs) of fMRI signals for a given target trial were estimated using sparse multivariate Bayesian parameter estimation (MVBPE), where sparsity constraint was used as a spatial feature selection. The classification was carried out by comparing estimated log evidences for the two competing models. The MVBPE to decode brain states from hemodynamic responses (temporal) across the brain (spatial) seeks to find an optimal set of voxels (features) with different weights to best represent observed signals. Note that decoding is an ill-posed problem in reducing BOLD signals obtained from many voxels to a binarized or multiclass label of brain states. To resolve this ill-posed regression problem in terms of a Bayesian framework, researchers have used different priors based on various encoding hypotheses to estimate voxel weights. In this study, we used MVBPE with a distributed sparse constraint, which plays an important role in online feature selection during model parameter estimation in MVBPE.

Having too many features despite the small sample data generally cause an overfitting problem, called ‘the curse of dimensionality’. As a method to overcome the overfitting problem in the classification, feature selection controls the number of voxels (or features) relative to the number of samples (or scans) [33], although a greater number of weakly informative voxels may be useful in discriminating brain states in some cases [34]. In this study, among the 39,457 ± 23,384 voxels (mean ± standard deviation) across subjects in the initial feature masks, only 1,940 ± 240 voxels were chosen during MVBPE. Since the SVM classifier in this study utilized a similar number of features (2,000) after RFE, the reduced number of features is not the only reason for the higher performance of the MVB over other classification methods. As explained in the above, the high accuracy of the sparse MVB may be attributed to the sparsity constraint, which represents distributed encoding of neural responses. This has largely been evidenced in previous studies that demonstrated the superior performance of a sparse model over a clustered model [12, 13, 15, 16].

In addition to the sparse constraint, there are several advantages of the proposed MVB over other methods, particularly SVM in the feature selection. Due to separation of feature selection (RFE in this study) and model optimization, it is not trivial to find a better method of feature selection in SVM before optimizing a classification model. In contrast, the MVB classifier includes controlling the weights of voxels (feature selection process) as a part of classification model building. Furthermore, general feature selection in the SVM, such as the searchlight approach, utilizes univariate or localized information to determine feature mask while sparse MVB utilizes multivariate features and in particular, spatially distributed information.

In this study, the classification performance was significantly improved by testing two events of the same class compared with testing a single event. Although three trials showed further improvement, two trials in a class resulted in sufficiently improved classification performance. Information from more temporal profiles is advantageous in the model optimization for highly noisy and variable fMRI data. An additional advantage of the proposed method is that it can be easily extended to multiple trials.

We note that ISI is an important factor for better classification of each single event. The significant correlation of ISI with the free energy approximation (log model evidence) suggests that the appropriate positioning of the target stimuli with regard to longer ISI, especially the post-stimulus interval, can improve classification performance. This appropriate positioning will reduce temporal contamination by a consecutive event, which is unavoidable in the rapid event-related design [33–35] and aggravates classification performance [36]. This is a very crucial aspect in the design of stimulus presentation for practical purposes, such as forensic cases, as in this study.

The current study is designed for application to forensic investigation, for the purpose of identifying an individual’s memory retrieval of a voluntary action. The basic scheme is that the voluntary action will be easily or spontaneously retrieved when a cue is presented. Note that memory retrieval process differs across individuals as shown in Fig 6. Although previous studies in groups demonstrated increased activation in various brain regions such as the prefrontal cortex [37, 38], limbic areas [39], and anterior cingulate cortex and superior frontal gyrus [40], the group results may not be directly used for the classification of individual responses. Therefore, we proposed a framework for localizing brain involvement for individualized memory retrieval process using a stimulatory experiment (real practice with a video recording), in which real target stimuli can be embedded with stimuli from the simulation task without being recognizable. The individualized localization results of brain involvement for the memory retrieval process are subsequently used for feature selection, which is known to enhance fMRI-based deception detection performance [41].

Although we used stimuli in the stimulatory experiment as target stimuli for the purpose of evaluation, we can replace these target stimuli with real case stimuli; for example, the photos used in an interrogation may conform to a similar format as the photos from this experiment. By conducting a “saw” and “didn’t see” task rather than an “I did” or “I didn’t do” task in the fMRI, we avoided participant recognition of the hidden intention of the task, which is a good strategy for a situation in which subjects may attempt to hide their memory retrieval process. While participants spontaneously identify “saw” or “didn’t see” for few real case stimuli among many experimental stimuli, the current method will extract the voluntariness of the past event by decoding differential memory retrieval processes. An additional strength of the proposed framework is the capacity to evaluate the accuracy (confidence level) of the classifier for each individual using a leave-one-out evaluation of known targets. For example, S16 and S17 in Table 1 showed low classification accuracy. Thus, we can decide not to use the current method for those subjects in practice, which is a very important requirement in forensic applications. By evaluating classification accuracy for each individual, we can estimate whether the classification outcome should be considered or rejected as evidence in the interrogation. In addition to classification, since the free energy is an approximation to the model evidence, the two-model MVB approach could be used to produce posterior probabilities of each trial that falls in a particular category. Although we used classification to ensure comparability with the SVM in this study, a probabilistic approach could be useful in practical applications of current MVB approach.

In conclusion, we proposed a framework for classifying responses to few unknown stimuli among many known stimuli presented as single or several target trials in the rapid event-related design. We used a multivariate Bayesian approach, which included spatio-temporal information in the classification of single or multiple trials and showed higher classification performance compared to the SVM classification. The proposed method can decode subject-specific memory retrieval of voluntary behavior and may be useful for reliable lie detection in more natural environments using fMRI.
